# Immunomodulatory roles of CTRP3 in endotoxemia and metabolic stress

**DOI:** 10.14814/phy2.12735

**Published:** 2016-03-20

**Authors:** Pia S. Petersen, Risa M. Wolf, Xia Lei, Jonathan M. Peterson, G. William Wong

**Affiliations:** ^1^ Department of PhysiologyThe Johns Hopkins University School of MedicineBaltimoreMaryland; ^2^ Center for Metabolism and Obesity ResearchThe Johns Hopkins University School of MedicineBaltimoreMaryland; ^3^ Department of PediatricsThe Johns Hopkins University School of MedicineBaltimoreMaryland; ^4^ Present address: Department of Health SciencesCollege of Public HealthEast Tennessee State UniversityJohnson CityTN37601USA

**Keywords:** CTRP3, cytokines, inflammation, LPS, obesity

## Abstract

C1q/TNF‐related protein 3 (CTRP3) is a secreted hormone that modulates hepatic glucose and lipid metabolism. Its circulating levels are reduced in human and rodent models of obesity, a metabolic state accompanied by chronic low‐grade inflammation. Recent studies have demonstrated an anti‐inflammatory role for recombinant CTRP3 in attenuating LPS‐induced systemic inflammation, and its deficiency markedly exacerbates inflammation in a mouse model of rheumatoid arthritis. We used genetic mouse models to explore the immunomodulatory function of CTRP3 in response to acute (LPS challenge) and chronic (high‐fat diet) inflammatory stimuli. In a sublethal dose of LPS challenge, neither CTRP3 deficiency nor its overexpression in transgenic mice had an impact on IL‐1*β*, IL‐6, TNF‐*α*, or MIP‐2 induction at the serum protein or mRNA levels, contrary to previous findings based on recombinant CTRP3 administration. In a metabolic context, we measured 71 serum cytokine levels in wild‐type and CTRP3 transgenic mice fed a high‐fat diet or a matched control low‐fat diet. On a low‐fat diet, CTRP3 transgenic mice had elevated circulating levels of multiple chemokines (CCL11, CXCL9, CXCL10, CCL17, CX3CL1, CCL22 and sCD30). However, when obesity was induced with a high‐fat diet, CTRP3 transgenic mice had lower circulating levels of IL‐5, TNF‐*α*, sVEGF2, and sVEGFR3, and a higher level of soluble gp130. Contingent upon the metabolic state, CTRP3 overexpression altered chemokine levels in lean mice, and attenuated systemic inflammation in the setting of obesity and insulin resistance. These results highlight a context‐dependent immunomodulatory role for CTRP3.

## Introduction

CTRP3 is a member of the C1q/TNF‐related protein (CTRP) family and was originally identified based on its sequence homology to adiponectin (Wong et al. 2004). Adiponectin, a widely studied insulin‐sensitizing adipokine, and CTRPs belong to the larger C1q family of proteins that share a signature C‐terminal globular domain homologous to the immune complement C1q (Kishore et al. 2004; Seldin et al. 2014). CTRP3, also known as CORS26 (Maeda et al. 2001), is expressed by a variety of tissues and cell types with the highest expression seen in adipose tissue, kidney, uterus, and testis in adult animals (Wong et al. 2008).

Relatively little is known about the physiological function of CTRP3. Circulating levels of CTRP3 have been found to decrease in rodent models of obesity and diabetes (Peterson et al. 2010; Li et al. 2014), as well as in humans with obesity (Wolf et al. 2015b), metabolic syndrome (Yoo et al. 2013), type 2 diabetes (Ban et al. 2014), and obese individuals with hypertension and insulin resistance (Deng et al. 2015). Women with polycystic ovarian syndrome also have lower serum CTRP3 levels that are increased by metformin treatment (Tan et al. 2013). Functional studies using recombinant protein infusion and transgenic (Tg) overexpression in mice have demonstrated an important role for CTRP3 in regulating hepatic gluconeogenesis (Peterson et al. 2010) and lipid metabolism (Peterson et al. 2013). Its deficiency in *Ctrp3* knockout (KO) mice, however, results in a marked reduction in liver size in response to high‐fat feeding without overt defects in whole‐body energy balance or glucose homeostasis (Wolf et al. 2015a). In addition, we have observed altered circulating levels of proinflammatory IL‐6 and profibrotic TGF‐*β* in CTRP3 KO mice consuming a high‐fat diet (Wolf et al. 2015a).

Interestingly, overexpression of CTRP3 in mice has been shown to confer protection against ischemic heart attack (Yi et al. 2012), but adenoviral‐mediated overexpression of CTRP3 in the abdominal or carotid arteries of a rat model of adenine diet‐induced chronic renal failure appears to promote calcification of the abdominal aorta and arterial ring (Zhou et al. 2014). These studies are based on protein overexpression, and the impact of CTRP3 deficiency on cardiovascular function remains unknown. However, CTRP3 has also recently been shown to have anti‐inflammatory properties in vitro (Kopp et al. 2010a,b; Hofmann et al. 2011), ex vivo (Kopp et al. 2010a), and in vivo (Murayama et al. 2014; Schmid et al. 2014). More specifically, mice deficient in CTRP3 have a greatly exacerbated inflammatory joint pathology in a collagen‐induced rheumatoid arthritis model (Murayama et al. 2014), and recombinant CTRP3 administration was found to attenuate systemic inflammation in wild‐type mice challenged with a sublethal dose of bacterial‐derived lipopolysaccharide (LPS) (Schmid et al. 2014).

As a consequence of macrophage infiltration into white adipose tissue and the array of proinflammatory cytokines they secrete (Weisberg et al. 2003; Xu et al. 2003; Kanda et al. 2006; Harman‐Boehm et al. 2007), a state of chronic low‐grade inflammation is prevalent in human and rodent models of obesity (Hotamisligil 2006). Remarkably, other immune cell types, such as T cells (Feuerer et al. 2009), B cells (DeFuria et al. 2013), neutrophils (Talukdar et al. 2012), mast cells (Liu et al. 2009), and eosinophils (Wu et al. 2011), residing in or being recruited to the fat pad also play critical immunomodulatory roles in maintaining adipose tissue health under normal and obese states. Given that adipose tissue secretes large numbers of adipokines that affect systemic insulin sensitivity and energy metabolism (Rosen and Spiegelman 2006, 2014), the immune‐metabolic axis takes on a systemic metabolic effect (Odegaard and Chawla 2013).

Using gain‐ and loss‐of‐function mouse models of CTRP3 (Peterson et al. 2013; Wolf et al. 2015a), we explored the role of CTRP3 in the inflammatory response induced by LPS challenge and high‐fat feeding. LPS challenge is representative of an acute inflammatory stimuli mimicking bacterial infection, while high‐fat feeding induces a chronic, low‐grade, inflammatory state. We provided genetic evidence that CTRP3 modulates circulating cytokine levels in a diet and metabolic state‐dependent manner, but appears to play minimal role in LPS‐induced systemic inflammation.

## Materials and Methods

### Experimental animals

CTRP3 transgenic (Tg) male mice (with a C57BL/6J genetic background) (Peterson et al. 2013), CTRP3 KO male mice (C57BL/6J genetic background) (Wolf et al. 2015a), and WT littermate controls were housed in polycarbonate cages on a 12‐h light–dark photocycle with ad libitum access to water and food. Mice were fed a standard laboratory chow diet (chow, 18% kcal from fat, 2018SX; Teklad Global Rodent Diets). All animal protocols were approved by the Institutional Animal Care and Use Committee of The Johns Hopkins University School of Medicine.

### Diet‐induced obese mouse model

CTRP3 Tg and control littermate WT male mice were fed either a high‐fat diet (HFD; 60% kcal derived from fat, Research Diets; D12492) or the matched control low‐fat diet (LFD; 10% kcal derived from fat, Research Diets; D12450B). Experimental diets were provided for a period of 14 weeks, beginning at 4 weeks of age. Mice were fasted overnight before the collection of blood samples.

### LPS administration

Male mice (10–14 weeks old) were fasted overnight and given an intraperitoneal injection of 1 *μ*g LPS (from *Escherichia coli* strain 055:B5; Sigma) in 50 *μ*L saline. Control mice were injected with saline only. The LPS dose was chosen based on a previous study in which 1 *μ*g LPS per mouse was sufficient to robustly induce inflammatory cytokine gene expression and increase serum levels in mice without causing an overwhelming inflammatory response that might override the anti‐inflammatory effects of CTRP3 (Schmid et al. 2014). Two hours later, a blood sample was collected via tail vein bleed. Mice were euthanized and the gonadal (epididymal) white adipose tissue was collected and snap frozen in liquid nitrogen. Tissue samples were stored at −80°C until further analysis.

### Quantitative real‐time PCR

Adipose RNA was isolated with Tripure Isolation Reagent (Roche) and reverse‐transcribed with the GoScript^™^ reverse transcription system (Promega). Quantitative real‐time PCR was performed on the CFX Connect Real Time System (Bio‐Rad) with iTaq Universal SYBR Green Supermix (Bio‐Rad). Relative levels of mRNA were calculated using the ΔΔCt method with peptidylprolyl isomerase A (also known as cyclophilin A) as a reference gene (Schmittgen and Livak 2008). There was no significant difference between the mean CT values for cyclophilin A between groups. The sequences of the primers used were: cyclophilin A (*CypA*): forward 5′‐AGCACTGGGGAGAAAGGATT‐3′ and reverse 5′‐CATGCCTTCTTTCACCTTCC‐3′; *Ctrp3*: forward 5′‐CATCTGGTGGCACCTGCTG‐3′ and reverse 5′‐TGACACAGGCAAAATGGGAG‐3′; *Il‐1β*: forward 5′‐GCCACCTTTTGACAGTGATGA‐3′ and reverse 5′‐GACAGCCCAGGTCAAAGGTT‐3′; *ll‐6*: forward 5′‐TTCCATCCAGTTGCCTTCTTG‐3′ and reverse 5′‐GAAGGCCGTGGTTGTCACC‐3′; *Cxcl‐2*: forward 5′‐TCCAGAGCTTGAGTGTGACG‐3′ and reverse 5′‐AGGCACATCAGGTACGATCC‐3′.

### Measurement of cytokine and chemokine levels

Mouse serum was harvested by tail bleed and at the time of euthanasia (~14 weeks old). Serum samples were separated by a Microvette^®^ CB 300 (Sarstedt, Nümbrecht, Germany) and centrifuged at 10,000 *g* for 5 min. TNF‐*α* (Millipore), IL‐1*β* (R&D Systems), and IL‐6 (Abcam) levels were determined using commercially available ELISA kits. Due to high circulating levels of these cytokines, samples had to be diluted 1:50 for the IL‐1*β* and IL‐6 assays, and 1:25 for the TNF‐*α* assay kit to meet the limits of the standard curve. For IL‐6 and IL‐1*β*, we included samples from mice treated with saline alone; however, the serum levels of TNF‐*α* in the saline‐injected control mice were below the detection limit of the standard curve and therefore its values were set at the detection limit of 2.3 ng/mL.

### Multiplex cytokine profiling

Cytokine profiling of WT and Tg mice fed a low‐fat or a high‐fat diet was carried out as described previously (Petersen et al. 2014). In brief, mouse blood samples (~200 *μ*L) were collected by tail vein bleed and separated by a Microvette^®^ CB 300 (SARSTEDT, Numbrecht, Germany). Serum cytokine levels (*N* = 8–9 per group) were measured in a Luminex Instrument (Luminex, Austin, TX) using a multiplex bead‐based assay (EMD Millipore, Billerica, MA) and analyzed by XPonent 3.1 Software (Millipore, Billerica, MA). Five separate multiplex assays, based on the known dynamic range of each cytokine, were carried out to cover a total of 71 cytokines. Some of the cytokines’ receptors are synthesized in membrane‐bound form, and proteolytic cleavage generates a soluble version that circulates in plasma. Thus, sCD30, sIL‐1RI, sIL‐1RII, sIL‐2Ra, sIL‐4R, sIL‐6R, sTNFRI, sTNFRII, sVEGFR1, sVEGFR2, sVEGFR3, sgp130, and sRAGE were also measured as part of the 71 cytokines profiled. Standards were provided for each mouse cytokine, from which standard curves were generated. Concentrations were determined for each of the 71 mouse cytokines relative to an appropriate 6‐point regression standard curve in which the mean fluorescence for each cytokine standard was transformed into known concentrations (pg/mL or ng/mL). Any sample below the detection limit of the assay (3.2 pg/ml) was excluded in analysis.

### Statistical analysis

For the LPS study, we performed one‐way ANOVA with Tukey's multiple comparison tests. The nonparametric Mann–Whitney *U* test was used on serum cytokine profiling data that did not pass the D'Agostino and Pearson omnibus normality tests. Differences between groups were considered to be significant when *P* < 0.05. For all statistical comparisons, * indicates *P *< 0.05; ** indicates *P *< 0.01; and *** indicates *P *< 0.005. All data are presented as mean ± standard error of the mean (SEM).

## Results

### CTRP3 expression does not impact inflammatory cytokine gene expression in response to LPS challenge

**Figure 1:**
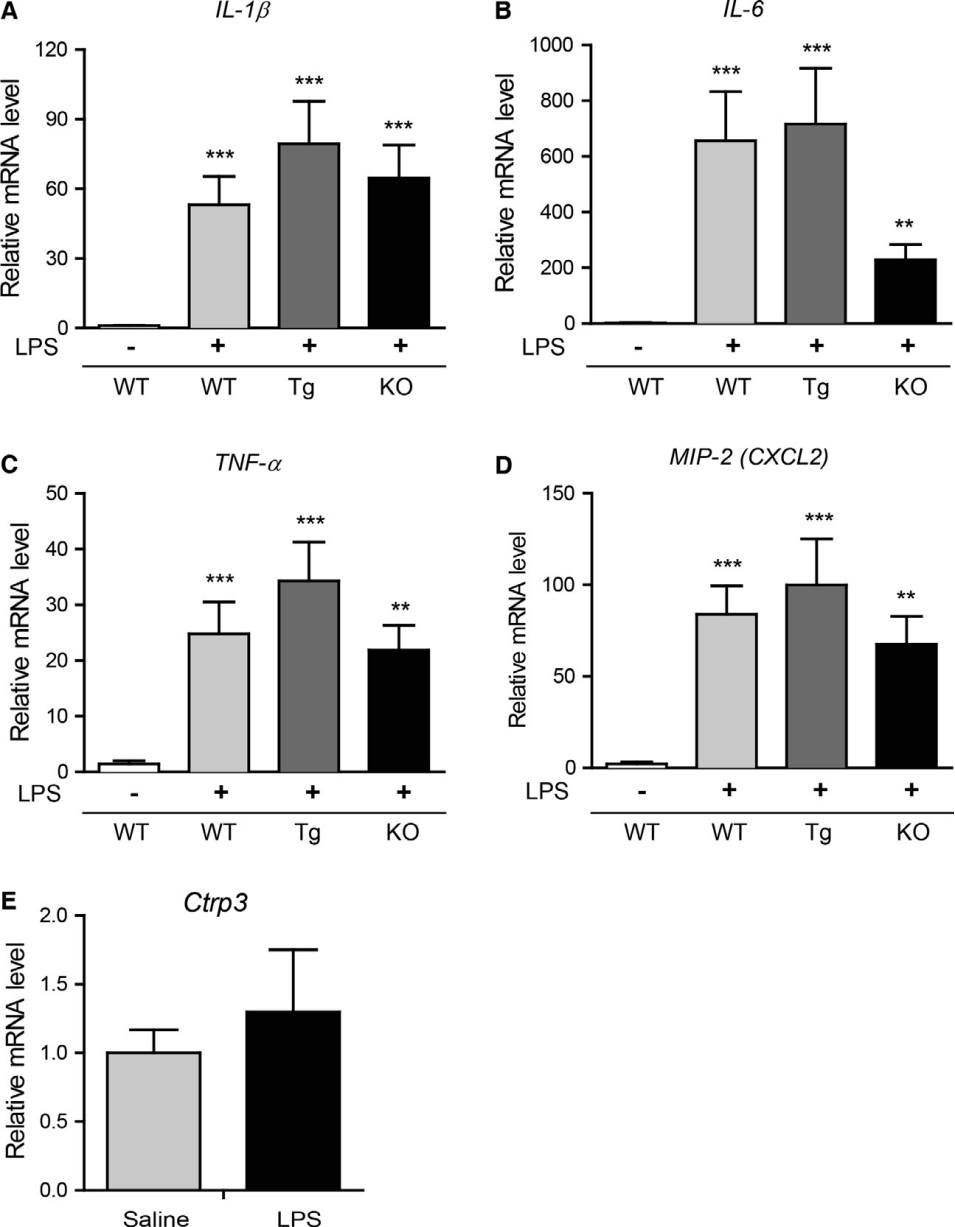
Increased expression of inflammatory cytokines in the epididymal fat depot of WT, CTRP3 Tg, and KO mice challenged with LPS. Quantitative real‐time PCR analysis of *Il1β* (A), *Il6* (B), *Tnfα* (C), and *Mip2/Cxcl2* (D) in the visceral (epididymal) fat depot of WT (*n* = 7), CTRP3 Tg (*n* = 8), and CTRP3 KO (*n* = 6) male mice injected with saline control or LPS (1 *μ*g). (E) Expression of *Ctrp3* in the visceral adipose tissue of LPS‐injected WT male mice. Expression levels were normalized to cyclophilin A (*CypA*). All data are expressed as mean ± SEM. *P < 0.05; **P < 0.01; ***P < 0.005 (saline vs. LPS injected mice)

Control wild‐type mice injected with saline had very low adipose expression of *IL‐1β*,* IL‐6*,* TNF‐α*, and *MIP‐2* (*CXCL2*) transcripts (Fig. 1). These are the cytokines known to be robustly induced by inflammatory stimuli such as bacteria‐derived LPS. Intraperitoneal injection of a sublethal dose of LPS significantly increased *IL‐1β*,* IL‐6*,* TNF‐α*, and *CXCL2* gene expression in the visceral (epididymal) adipose tissue of WT mice compared to control mice injected with saline only (Fig. 1A–D). LPS administration, however, did not affect the expression of *Ctrp3* mRNA in gonadal adipose tissue (Fig. 1E). In Tg mice with elevated circulating levels of CTRP3, as well as KO mice that lack CTRP3, LPS administration also increased the expression of *IL‐1β*,* IL‐6*,* TNF‐α*, and *CXCL2* mRNA relative to saline‐injected controls (Fig. 1A–D). Although *IL‐6* mRNA was induced by LPS in *Ctrp3*‐KO mice relative to saline‐injected control (*P *< 0.05 by Student's *t* test), it fell short of statistical significance when one‐way ANOVA with Tukey's multiple comparison tests were performed. Overall, our results did not show any statistically significant differences in the magnitude of inflammatory cytokine induction between the WT, Tg, and KO mice in response to LPS challenge.

### CTRP3 expression does not impact circulating cytokine levels in response to LPS challenge

**Figure 2:**
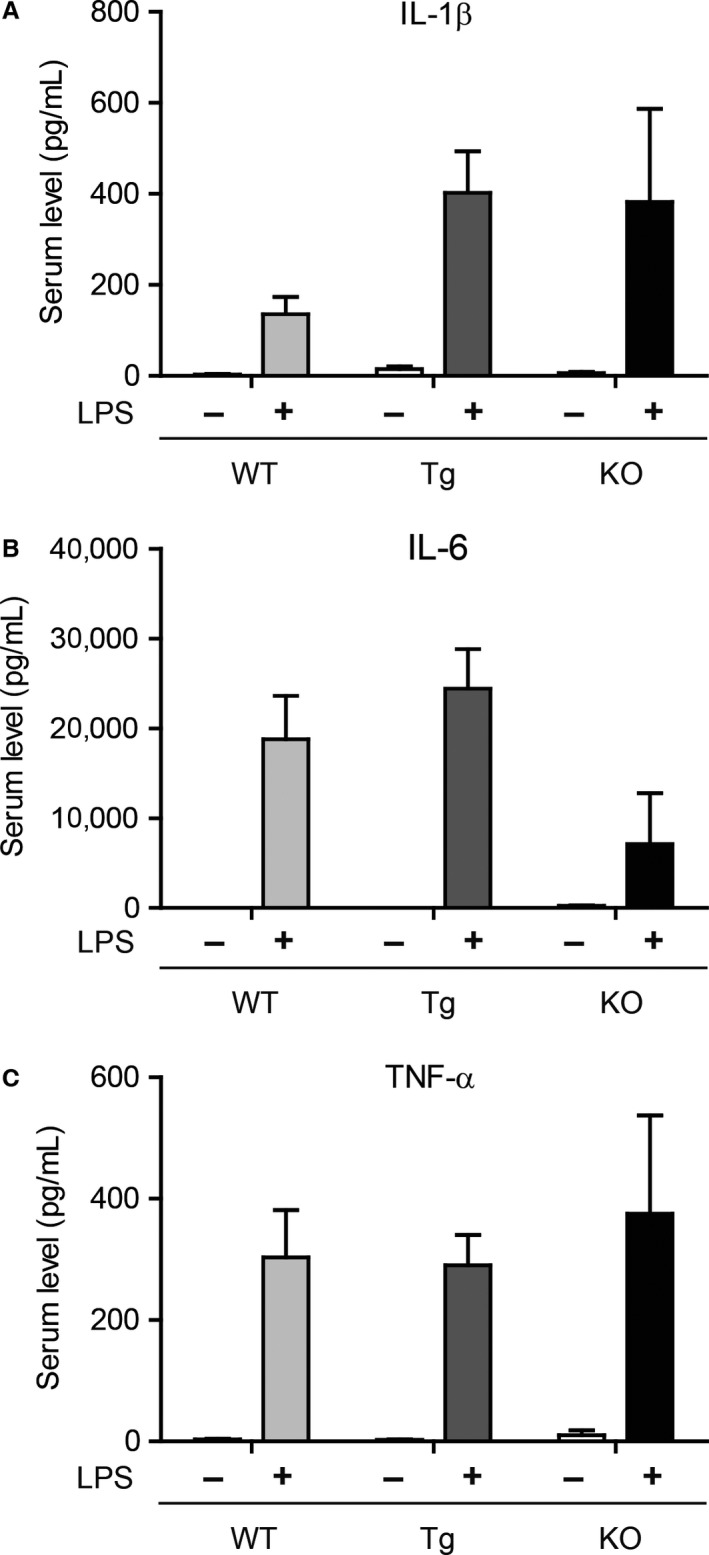
Increased serum levels of inflammatory cytokines in WT, CTRP3 Tg, and KO mice challenged with LPS. Serum IL‐1*β* (A), IL‐6 (B), and TNF‐*α* (C) levels in WT (*n* = 7), CTRP3 Tg (*n* = 8), and CTRP3 KO (*n* = 6) male mice injected with saline control or LPS (1 *μ*g). All data are expressed as mean ± SEM.

Since cytokine levels are regulated by both transcriptional and posttranscriptional mechanisms (Anderson 2008), we examined serum levels of IL‐1*β*, IL‐6, and TNF‐*α* in LPS‐injected animals in addition to gene expression. In accordance with the mRNA expression in visceral adipose tissue (Fig. 1), circulating levels of IL‐1*β*, IL‐6, and TNF‐*α* were also significantly elevated in LPS‐injected WT mice compared to saline‐injected controls (Fig. 2). In both CTRP3 Tg and KO mice injected with LPS, we also observed a significant increase in circulating levels of IL‐1*β*, IL‐6, and TNF‐*α* relative to saline‐injected WT controls (Fig. 2); the magnitude of change was comparable to, and not statistically different from, LPS‐injected WT mice. Importantly, in the absence of LPS exposure, the basal serum levels of IL‐1*β*, IL‐6, and TNF‐*α* were not different between CTRP3 Tg mice and WT controls (Table 1), nor was IL‐1*β* and TNF‐*α* different between *Ctrp3* KO mice and littermate controls (Wolf et al. 2015a).

**Table 1:** Summary of serum cytokine profiling in lean WT (*N* = 8) and CTRP3 Tg (*N* = 9) male mice fed a low‐fat diet. Except for A2M, adipsin, AGP, haptoglobin, SAP, PTX3, and SAA‐3 (ng/mL), all values are presented as pg/mL. *P < 0.05

Cytokine	WT	CTRP3 Tg	*P* value	Cytokine	WT	CTRP3 Tg	*P* value
Cytokines							
sCD30*	40 ± 6	19 ± 4	0.015	IL‐16	5591 ± 895	8261 ± 2271	0.3
sgp130	93 ± 37	311 ± 197	0.38	IL‐17	4.8 ± 1	5.9 ± 2	0.7
IL‐1*α*	518 ± 144	716 ± 81	0.23	IL‐22	7.8 ± 3	8.2 ± 4	0.9
IL‐1*β*	32 ± 14	15 ± 6	0.26	IL‐25	1424 ± 907	307 ± 65	0.3
IL‐2	1.6 ± 0.5	2.8 ± 1	0.47	IL‐27	55 ± 31	50 ± 29	0.9
IL‐4	2.2 ± 1	3.5 ± 1	0.37	IL‐28B	279 ± 23	275 ± 31	0.9
IL‐5	13 ± 3	11 ± 2	0.65	sIL‐1RI	1831 ± 233	1275 ± 337	0.2
IL‐6	14 ± 3	13 ± 2	0.72	sIL‐1RII	9931 ± 282	7143 ± 1501	0.1
IL‐7	6.5 ± 2	15 ± 4	0.27	sIL‐2R*α*	195 ± 17	178 ± 33	0.6
IL‐9	68 ± 28	77 ± 28	0.86	sIL‐4R	2433 ± 402	1394 ± 379	0.2
IL‐10	12 ± 3	15 ± 5	0.56	sIL‐6R	9069 ± 682	6487 ± 1569	0.2
IL12p40	48 ± 29	6.1 ± 2	0.38	PTX‐3	11 ± 1	8.9 ± 1	0.2
IL‐12p70	55 ± 21	39 ± 13	0.53	TNF‐*α*	4.05 ± 1	2.9 ± 0.4	0.3
IL‐13	115 ± 42	105 ± 15	0.82	sTNFRI	4376 ± 439	3169 ± 742	0.2
IL‐15	62 ± 36	80 ± 28	0.76	sTNFRII	3417 ± 389	2280 ± 531	0.1
Chemokines							
CCL2	51 ± 13	47 ± 13	0.83	CCL21	1880 ± 349	2975 ± 375	0.1
CCL3	79 ± 17	80 ± 14	0.99	CCL22*	13 ± 2	18 ± 2	0.014
CCL4	81 ± 23	93 ± 25	0.80	CXCL1	55 ± 9	66 ± 14	0.6
CCL5	24 ± 6	20 ± 5	0.66	CXCL2	148 ± 42	109 ± 13	0.4
CCL11**	539 ± 15	608 ± 10	0.0019	CXCL5*	8808 ± 962	11838 ± 1219	0.04
CCL12	46 ± 7	58 ± 7	0.29	CXCL9*	50 ± 7	76 ± 7	0.01
CCL17*	49 ± 8	91 ± 17	0.029	CXCL10*	108 ± 12	145 ± 11	0.03
CCL20	43 ± 8	29 ± 5	0.16	CX3CL1**	1257 ± 152	2304 ± 367	0.003
Growth and differentiation factors							
G‐CSF	473 ± 152	408 ± 68	0.69	VEGF	2 ± 1	1.8 ± 1	0.8
GM‐CSF	57 ± 19	40 ± 11	0.46	sVEGFR1	2063 ± 343	1430 ± 416	0.3
M‐CSF	57 ± 45	42 ± 14	0.77	sVEGFR2	31243 ± 2387	24597 ± 5740	0.4
TIMP‐1	429 ± 157	334 ± 89	0.58	sVEGFR3	28085 ± 1224	24772 ± 4512	0.5
Other cytokines							
sRAGE	82 ± 41	29 ± 8	0.17	AGP	0.03 ± 0.003	0.02 ± 0.003	0.1
Lipocalin2	23 ± 3	22 ± 4	0.80	A2M	314 ± 22	338 ± 20	0.4
SAA‐3	202 ± 13	292 ± 124	0.51	Haptoglobin	12 ± 2	15 ± 3	0.4
Adipsin	18 ± 1	19 ± 0.4	0.73	SAP	1362 ± 319	1601 ± 657	0.8
IFN‐g	19 ± 8	24 ± 9	0.82	EPO	339 ± 124	628 ± 171	0.2

### CTRP3 overexpression alters circulating cytokine levels in different metabolic context

**Figure 3:**
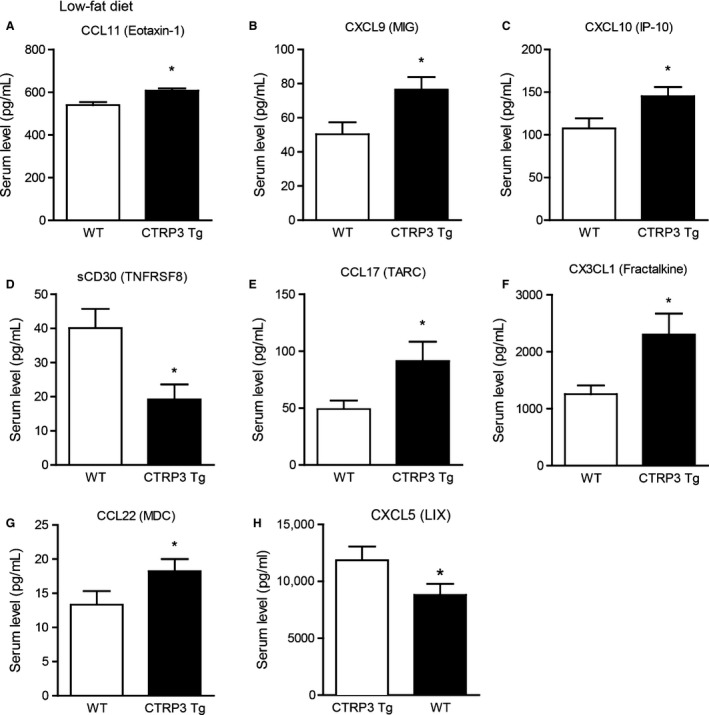
Altered serum cytokine levels in CTRP3 Tg compared to WT mice fed a control low‐fat diet. Serum levels of CCL11 (A), CXCL9 (B), CXCL10 (C), secreted CD30 (D), CCL17 (E), CX3CL1 (F), CCL22 (G) and CXCL5 (H) in WT and CTRP3 Tg male mice fed a high‐fat diet. All data are expressed as mean ± SEM (*N* = 8–9 per group). *P < 0.05.

**Figure 4:**
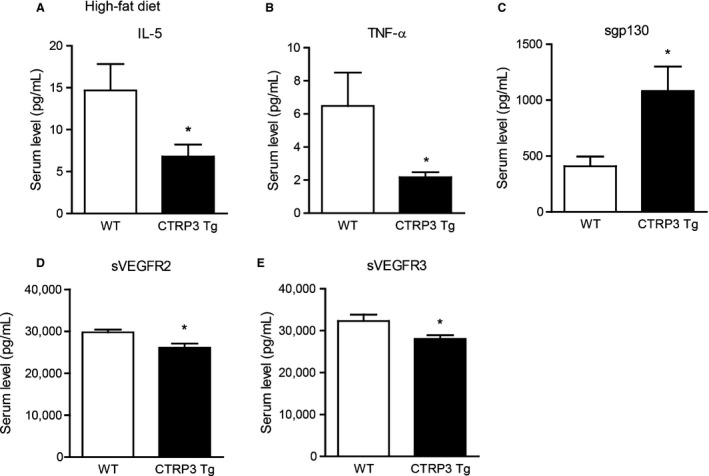
Altered serum cytokine levels in CTRP3 Tg compared to WT mice fed calorie‐dense high‐fat diet. Serum levels of IL‐5 (A), TNF‐*α* (B), sgp130 (C), secreted VEGF2 (D), and secreted VEGFR3 (E) in WT and CTRP3 Tg fed a low‐fat diet. All data are expressed as mean ± SEM (*N* = 8–9 per group). *P < 0.05.

**Figure 5:**
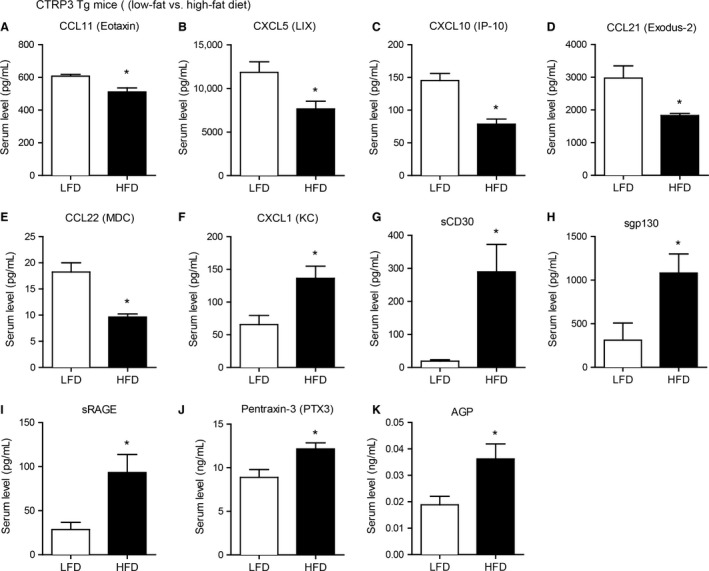
Altered serum cytokine levels in CTRP3 Tg fed a control low‐fat diet or a calorie‐dense high‐fat diet. Serum levels of CCL11 (A), CXCL5 (B), CXCL10 (C), CCL21 (D), CCL22 (E), CXCL1 (F), sCD30 (G), sgp130 (H), sRAGE (I), Pentraxin‐3 (J), and AGP (K) in CTRP3 Tg fed a low‐fat diet (LFD) or high‐fat diet (HFD). All data are expressed as mean ± SEM (*N* = 8–9 per group). *P < 0.05.

Circulating levels of many cytokines are altered by metabolic stress induced by high‐fat feeding (Petersen et al. 2014), and diet‐induced obesity frequently results in chronic inflammation (Weisberg et al. 2003; Hotamisligil 2006; Kanda et al. 2006; Harman‐Boehm et al. 2007). We examined the impact of CTRP3 overexpression on circulating cytokine and chemokine levels in WT and Tg mice fed a control low‐fat diet (LFD) or a calorie‐dense high‐fat diet (HFD) for 14 weeks. As expected, high‐fat feeding significantly increased the body weight of both WT and Tg mice to the same extent, and the body weights of the LFD‐fed WT and Tg mice were comparable (Peterson et al. 2013). Using a multiplex bead‐based assay approach (Dupont et al. 2005; Tighe et al. 2013; Petersen et al. 2014; Khalifian et al. 2015), we quantified the circulating levels of 71 cytokines, chemokines, and secreted cytokine receptors in both LFD‐fed and HFD‐fed mice (Tables 1 and 2). While >100 cytokines have been described thus far, the set of 71 cytokines, chemokines, and secreted cytokine receptors we chose to examine covers the major cytokines and includes diverse immune and nonimmune functions. For CTRP3 Tg mice fed a control LFD, we observed higher circulating levels of multiple chemokines – CCL11 (Eotaxin‐1), CXCL9 (MIG), CXCL10 (IP‐10), CCL17 (TARC), CX3CL1 (Fractalkine), and CCL22 (MDC) – and lower levels of secreted CD30 compared to WT littermate controls (Fig. 3). When subjected to high‐fat feeding, CTRP3 Tg mice had significantly lower circulating levels of IL‐5, TNF‐*α*, secreted VEGF2, and secreted VEGFR3, and significantly higher levels of soluble gp130 compared to WT littermate controls (Fig. 4). To assess the potential interaction between genes and environments, we compared serum cytokine levels in CTRP3 Tg mice fed either a control LFD or a calorie‐dense HFD. Depending on the diet, CTRP3 overexpression differentially modulated the circulating levels of CCL11 (Eotaxin‐1), CXCL5, CXCL10 (IP‐10), CCL21, CCL22 (MDC), CXCL1, sCD30, sgp130, sRAGE, pentraxin‐3 (PTX‐3), and AGP (Fig. 5).

**Table 2:** Summary of serum cytokine profiling in obese WT (*N* = 8) and CTRP3 Tg (*N* = 9) male mice fed a calorie‐dense high‐fat diet. Except for A2M, adipsin, AGP, haptoglobin, SAP, PTX3, and SAA‐3 (ng/mL), all other values are presented as pg/mL. *P < 0.05

Cytokine	WT	CTRP3 Tg	*P* value	Cytokine	WT	CTRP3 Tg	*P* value
Cytokines							
sCD30	86 ± 18	290 ± 93	0.1	IL‐17	7 ± 2	4 ± 1	0.3
sgp130*	409 ± 86	1082 ± 244	0.03	IL‐22	4 ± 1	4 ± 0.4	0.8
IL‐1*α*	256 ± 92	402 ± 193	0.5	IL‐25	396 ± 98	376 ± 143	0.9
IL‐1*β*	32 ± 15	10 ± 1	0.1	IL‐27	26 ± 6	22 ± 5	0.6
IL‐2	2 ± 1	3 ± 1	0.5	IL‐28B	129 ± 11	112 ± 22	0.5
IL‐5*	15 ± 3	7 ± 1	0.03	sIL‐1RI	918 ± 128	1406 ± 249	0.2
IL‐6	7 ± 2	11 ± 5	0.4	sIL‐1RII	8635 ± 360	8023 ± 257	0.2
IL‐9	104 ± 25	124 ± 49	0.7	sIL‐2R*α*	237 ± 15	214 ± 15	0.3
IL‐10	12 ± 3	12 ± 2	1.0	sIL‐4R	1744 ± 150	2087 ± 346	0.4
IL12p40	4 ± 1	3 ± 1	0.6	sIL‐6R	7584 ± 700	8195 ± 399	0.4
IL‐12p70	40 ± 15	15 ± 3	0.4	PTX‐3	11 ± 1	12 ± 1	0.3
IL‐13	67 ± 12	80 ± 12	0.4	TNF‐α*	6 ± 2	2 ± 0.4	0.04
IL‐15	16 ± 3	194 ± 71	0.4	sTNFRI	3846 ± 181	3882 ± 231	0.9
IL‐16	4392 ± 664	4973 ± 498	0.5	sTNFRII	3155 ± 305	3309 ± 155	0.6
Chemokines							
CCL2	55 ± 11	127 ± 86	0.4	CCL21	2005 ± 93	1830 ± 61	0.1
CCL3	59 ± 9	54 ± 9	0.6	CCL22	9 ± 0.4	10 ± 1	0.3
CCL4	34 ± 8	45 ± 8	0.4	CXCL1	140 ± 36	136 ± 21	0.9
CCL5	19 ± 3	15 ± 2	0.3	CXCL5	8148 ± 1316	7643 ± 969	0.7
CCL11	495 ± 20	511 ± 28	0.6	CXCL9	75 ± 7	73 ± 20	0.9
CCL12	54 ± 6	45 ± 3	0.2	CXCL10	98 ± 10	79 ± 9	0.1
CCL17	23 ± 5	23 ± 8	1.0	CX3CL1	1558 ± 104	1343 ± 104	0.2
Growth and differentiation factors							
G‐CSF	614 ± 120	908 ± 421	0.5	VEGF	4 ± 1	3 ± 1	0.4
GM‐CSF	36 ± 7	35 ± 10	1.0	sVEGFR1	1078 ± 104	925 ± 127	0.4
M‐CSF	1089 ± 503	344 ± 155	0.2	sVEGFR2*	29748 ± 621	26069 ± 989	0.02
TIMP‐1	335 ± 86	282 ± 55	0.6	sVEGFR3*	32269 ± 1556	28016 ± 677	0.02
Other cytokines							
sRAGE	112 ± 39	93 ± 17	0.6	AGP	0 ± 0.005	0 ± 0.01	0.7
Lipocalin2	16 ± 1	25 ± 6	0.1	A2M	334 ± 20	335 ± 24	1.0
SAA‐3	65 ± 11	87 ± 28	0.5	Haptoglobin	10 ± 2	10 ± 3	0.9
Adipsin	17 ± 1	19 ± 1	0.1	SAP	526 ± 64	1837 ± 814	0.1
IFN‐g	8.2 ± 2	14 ± 4	0.2	EPO	985 ± 338	675 ± 224	0.5

## Discussion

In light of recent studies in support of anti‐inflammatory properties for CTRP3 (Kopp et al. 2010a,b; Hofmann et al. 2011; Murayama et al. 2014; Schmid et al. 2014), we sought to further explore the immunomodulatory capabilities of CTRP3 using two mouse models, CTRP3 Tg and CTRP3 KO mice (Peterson et al. 2013; Wolf et al. 2015a). Surprisingly, in the context of both CTRP3 overexpression and deficiency, we did not observe any differences in the magnitude of proinflammatory IL‐1*β*, IL‐6, or TNF‐*α* induction in response to acute LPS challenge compared to WT controls. This contradicts a previous study by Schmid et al. in which recombinant CTRP3 administration suppressed inflammatory cytokine secretion upon LPS challenge (Schmid et al. 2014). Several other in vitro studies suggest that CTRP3 should antagonize LPS action by physically binding to Toll‐like receptor 4 (TLR4), blocking LPS from binding to its receptor (Compton and Cheatham 2010; Kopp et al. 2010a). How and which domain of CTRP3—the N‐terminus, collagen domain, or globular C1q domain—physically interacts with TLR4 is unknown, but presumably, CTRP3 binds to a site close to the LPS binding site on TLR4 in order to block LPS from binding to its receptor.

Intriguingly, the route of recombinant CTRP3 administration appears to have a significant impact on its ability to protect against LPS‐induced inflammation, as intraperitoneal, but not intravenous, injection of recombinant CTRP3 protects mice against systemic inflammation (Schmid et al. 2014). It was thought that the amount of recombinant protein (10 *μ*g) injected via intravenous route might not be sufficient to elicit an anti‐inflammatory effect, although the same dose was given intraperitoneally. In the present study, we chose to use the same dose of LPS and route of injection (intraperitoneal) as described by Schmid et al., given their success with CTRP3‐mediated cytokine suppression (Schmid et al. 2014). Our data did not support a role for CTRP3 in modulating an acute inflammatory response to bacteria‐derived LPS, at least not in the in vivo milieu where CTRP3 was entirely absent or its circulating level was chronically and substantially elevated (>fivefold) above physiological levels. It remains to be established whether CTRP3 overexpression or deficiency has an impact on the survival of mice in the presence of a higher, more likely lethal dose of LPS.

In contrast to the LPS injection study, we did observe an immunomodulatory function for CTRP3 in a metabolic context. We employed a multiplex bead‐based assay method to measure the serum levels of 71 cytokines, chemokines, secreted cytokine receptors, and acute phase proteins in lean and obese states. With this recent technological advancement, it is now possible to quantify large numbers of cytokines using only small, and often limited, amounts of mouse serum samples (Khalifian et al. 2015). Importantly, the multiplex assay is highly reproducible, with sensitivity comparable to traditional ELISA assays (Dupont et al. 2005; Tighe et al. 2013). Using this unbiased approach, we showed that CTRP3 Tg mice, when fed a control LFD, had reduced circulating levels of sCD30 and increased levels of seven chemokines—CCL11 (eotaxin‐1), CCL17 (TARC), CCL22 (MDC), CXCL9 (MIG), CXCL10 (IP‐10), CX3CL1 (fractalkine) and CXCL5 (LIX)—when compared to WT controls. These chemotactic factors are known to act on eosinophils (Garcia‐Zepeda et al. 1996), monocytes (Bazan et al. 1997; Godiska et al. 1997), NK cells (Godiska et al. 1997), macrophages (Imai et al. 1996), and lymphocytes (Taub et al. 1993; Liao et al. 1995; Bazan et al. 1997). The link between these chemokines and metabolism, however, is presently unclear since the metabolic profiles—body weight, glucose and insulin tolerance, serum lipid levels—were indistinguishable between WT and CTRP3 Tg mice fed a LFD (Peterson et al. 2013). It remains to be determined whether the immune response to specific pathogens is altered in CTRP3 Tg mice.

We examined the consequences of CTRP3 overexpression on circulating cytokines in diet‐induced obesity. In the obese state, CTRP3 overexpression altered systemic inflammation, as indicated by a reduction in serum IL‐5 and TNF‐*α*, and an increase in soluble gp130 (sgp130). TNF‐*α* is a potent inducer of insulin resistance (Hotamisligil et al. 1993) and its levels are increased in human obesity (Hotamisligil et al. 1995). IL‐5 is a major activator of eosinophil cells that participate in inflammation (Kouro and Takatsu 2009). In fact, serum levels of IL‐5 are also significantly elevated in human obesity (Schmidt et al. 2015). Soluble gp130 is known to antagonize inflammatory responses by binding to the IL‐6 family of cytokines (Silver and Hunter 2010), and its serum levels are increased in older individuals with metabolic syndrome (Zuliani et al. 2010). Thus, our data suggest that a chronic increase in plasma CTRP3 levels in transgenic mice attenuates systemic inflammation in response to high‐fat feeding, consistent with the improved insulin sensitivity seen in these animals (Peterson et al. 2013).

It is known that complex phenotypes, including many common polygenic diseases, arise from the interaction between genes and environments (Grarup and Andersen 2007; Franks 2011). The impact of CTRP3 overexpression may differ depending on environmental contexts. To assess this, we compared the circulating cytokine levels in mice fed either a control LFD or a calorie‐dense HFD. Depending on the diets, our results show that *Ctrp3* gene overexpression differentially modulates circulating cytokine levels. Notably, we observed reduced levels of multiple chemokines—CCL11 (eotaxin), CXCL5, CXCL10, CCL21 (exodus‐2), CCL22 (MDC)—in HFD‐fed CTRP3 Tg mice relative to the LFD‐fed group; these chemokines are known to play important roles in inflammation. In contrast, circulating levels of cytokines that correlate with reduced inflammation—secreted gp130 and secreted receptor for advanced glycation end products (sRAGE)—are increased in CTRP3 Tg mice fed a HFD relative to Tg animals fed a control LFD. Despite the robust changes seen in several circulating cytokines, additional studies are needed to distinguish whether these alterations are directly or indirectly linked to CTRP3 overexpression in two different dietary contexts, and the functional consequences for in vivo physiology.

In summary, based on gain‐ and loss‐of‐function mouse models, we found no evidence to support a role for CTRP3 in modulating acute LPS‐induced systemic inflammation. However, our study does identify an immunomodulatory role for CTRP3 in influencing chronic and systemic inflammation associated with diet‐induced obesity and insulin resistance.

## Conflict of Interest

None declared.
